# Mechanical and Physical Properties of Recycled-Carbon-Fiber-Reinforced Polylactide Fused Deposition Modelling Filament

**DOI:** 10.3390/ma15010190

**Published:** 2021-12-28

**Authors:** Nur’ain Wahidah Ya Omar, Norshah Aizat Shuaib, Mohd Haidiezul Jamal Ab Hadi, Azwan Iskandar Azmi, Muhamad Nur Misbah

**Affiliations:** 1 Faculty of Mechanical Engineering Technology, Universiti Malaysia Perlis (UniMAP), Arau 02600, Perlis, Malaysia; ainwahidah8077@gmail.com (N.W.Y.O.); haidiezul@unimap.edu.my (M.H.J.A.H.); azwaniskandar@unimap.edu.my (A.I.A.); mnur@unimap.edu.my (M.N.M.); 2 Centre of Excellence for Biomass Utilization, Universiti Malaysia Perlis (UniMAP), Arau 02600, Perlis, Malaysia

**Keywords:** recycled carbon fiber, fused deposition modelling, composite recycling, 3D printing, reinforced filament composite

## Abstract

Carbon-fiber-reinforced plastic materials have attracted several applications, including the fused deposition modelling (FDM) process. As a cheaper and more environmentally friendly alternative to its virgin counterpart, the use of milled recycled carbon fiber (rCF) has received much attention. The quality of the feed filament is important to avoid filament breakage and clogged nozzles during the FDM printing process. However, information about the effect of material parameters on the mechanical and physical properties of short rCF-reinforced FDM filament is still limited. This paper presents the effect of fiber loading (10 wt%, 20 wt%, and 30 wt%) and fiber size (63 µm, 75 µm, and 150 µm) on the filament’s tensile properties, surface roughness, microstructure, porosity level, density, and water absorptivity. The results show that the addition of 63 µm fibers at 10 wt% loading can enhance filament tensile properties with minimal surface roughness and porosity level. The addition of rCF increased the density and reduced the material’s water intake. This study also indicates a clear trade-off between the optimized properties. Hence, it is recommended that the optimization of rCF should consider the final application of the product. The findings of this study provide a new manufacturing strategy in utilizing milled rCF in potential 3D printing-based applications.

## 1. Introduction

With the growth of science and technology over recent decades, composite materials made of fiber-reinforced plastics (FRP) have started to receive vast attention as alternatives to those conventional materials [1]. The reinforcement, such as carbon, glass, or any natural fiber, can be embedded within low-density plastic to improve material strength and reduce the fabrication cost.

The global market for innovative products with complex designs and intricate detailing in industrial sectors, such as construction and transportation, has evolved through the usage of 3D printing technology [2]. Interest in additive manufacturing (AM) with thermoplastic material has increased rapidly on account of its low cost and does not require additional tooling and molding [3]. AM or 3D printing is a process of amalgamating materials, creating physical objects generated from a fitting process in Computer-Aided Design (CAD) [4]. This technology has the ability to fabricate structures with complex geometries with minimal aid, which is often impracticable with traditional manufacturing machines [5].

Among several 3D printing technologies available, fused deposition modelling (FDM) has been of particular interest due to its special ability to build fully functional parts with multi-materials of plastics [6]. FDM prints individual layers from the bottom to the top by heating and extruding thermoplastic filament. Low machine cost and ease of setting up the process parameters to produce a precise part have made this inventive technology an advantageous means for manufacturing in industries. However, several problems are identified related to this process. Improvement in terms of geometric stability, part quality, and product properties is essential for the FDM process. In addition, the performance of 3D printing is mostly governed by the feedstock or filament properties [7]. A proper understanding of these traits is crucial to achieving an efficient printing process.

Several findings in the literature have reported the effect of processing parameters in increasing the mechanical properties of FDM printed parts [8,9,10]. Even though selection and optimization process parameters affect the final product properties, the improvements are not substantial. The introduction of fibers into thermoplastic material to achieve optimum mechanical and functional properties is a promising solution to these problems [11].

Some studies have focused on the parameters used in the production of filaments with optimal diameter and homogeneity for the FDM method [12]. This knowledge would be important for the development of new filament materials. Despite that, achieving good interfacial adhesion between the fiber and the thermoplastic matrix in the FDM process is very challenging. As reported in the literature, fiber additives can be easily coagulated, which can to clogged nozzles, as well as non-uniformity, and porous filament [13]. To ensure superior performance of FDM product, the properties of the filaments need to be optimized through proper selection of particle size and weight loading of the reinforced fiber. Despite these issues, the number of studies focusing on parametric analysis of filament production is very limited. It is crucial to investigate optimum filament process parameters to ensure strong fiber-matrix interfaces, hence better product qualities.

The commonly applied reinforcement material in the FDM filament is short or continuous fibers. Generally, the high strength-to-weight ratio, stiffness, and corrosion resistance that can be attained from fiber reinforcement are inherited by these composites [13]. Several reinforcement materials have been introduced into the thermoplastic filaments, such as carbon nanotubes, graphene, copper, and carbon fiber. Carbon, glass, and Kevlar are known as thoroughly used high-performance fibers, while flax, basalt, wood, and bamboo are among popular natural fibers in the FDM composites industries [14]. The performance and defects of the filaments are influenced by both matrix and fiber.

One of the recent emerging fibers for the FDM process is recycled carbon fiber (rCF), as an alternative to virgin carbon fiber (vCF). From the literature, rCF which has been recovered from pyrolysis and chemical recycling methods has comparable performance with its virgin precursor and could be used an alternative material to vCF [15]. This is on account of the cheaper price of rCF as well as its excellent mechanical properties compared with its virgin counterpart. vCF typically has higher embodied energy (198–595 MJ/kg) compared to the energy demand in the recycling process of carbon fiber composites (0.27–90 MJ/kg) [16]. The advantages highlight the potential of rCF as a competitive alternative material to vCF. However, studies on the potential usage of rCF are rare as most studies focused on using vCF. 

The 3D printing of vCF-reinforced thermoplastic has already been documented in several studies [17,18,19,20]. Short carbon fibers are commonly used as reinforcement to enhance the properties of carbon fiber filament [21,22,23]. Previous studies have primarily concentrated on the development of filament composite materials for 3D printing. Comprehensive studies have been conducted on the incorporation of carbon fibers into the Acrylonitrile Butadiene Styrene (ABS) matrix for the development of innovative filaments [22,24,25]. There is also a growing interest in reinforcing polylactic acid (PLA) plastic with carbon fibers [26,27]. PLA is a biodegradable material made from renewable sources, which is ideal for environmental friendly application. Omar, et al. [28] reported that the incorporation of short or powdered carbon fiber in PLA samples can provide up to 350% improvement in terms of tensile strength, compared to only 150% and 23% in nylon and ABS, respectively.

Certain specifications need to be fulfilled for fiber-reinforced 3D printing filament in order for it to be processed by FDM. These include types of fiber and matrix, good fiber-to-matrix bonding, fiber homogeneity, and minimal porosity [29]. The requirement generally depends on the fiber length and fiber content.

Another important factor when considering a fiber-reinforced filament composite is fiber loading, because the mechanical performance of the composite is mainly affected by the reinforcement. There is a considerable variation, such as fiber distribution and interfacial bonding strength to achieve the optimum improvement of the mechanical properties. In general, high fiber content is needed to attain a high-performance filament composite. It is frequently noted that increased fiber content could lead to an incremental increase in the tensile properties. However, materials with higher fiber content can clog the nozzle during the printing process [30]. Besides that, a composite with higher fiber loading is difficult to fabricate into continuous filaments for FDM because of low toughness. Therefore, the properties of some final products of composite filament are limited by low fiber weights. Poor fiber wettability is expected, and increasing fiber weight would increase the porosity. Particle size is another important factor that has a significant effect on the properties of composite filaments. Different sizes of carbon fiber particles greatly modify surface characteristics and interface between carbon fiber and a thermoplastic matrix [31].

Compared to vCF, rCF is mostly used in the form of milled or short fiber as a result of shredding and cutting processes during the recycling procedure [15]. The addition of short fiber into the thermoplastic matrix has shown encouraging results in significantly improving the mechanical properties [18,32]. Nonetheless, the use of a fiber composite feedstock in 3D printers may be limited due to nozzle tip clogging caused by the agglomeration of the reinforcement material [33]. The addition can also cause porosity of the 3D printed products, resulting in a reduction of strength and possibly filament breakage during the printing process. To prevent this from happening, or at least reduce its rate of occurrence, the properties of the filament composite need to be optimized by achieving the best particle size and weight loading of the reinforced fiber. Despite these issues, the number of studies focusing on the optimized parameters in filament production is still low. Most studies are only based on the printed products, of which the final properties may have been affected by the selection of FDM processing parameters. Comprehension of how well the filament can accomplish its intended purpose and what developments can be made when reinforcement is applied is important is necessary for improvements to be made. To the current knowledge, there are limited extensive studies on the properties of rCF-reinforced thermoplastic polymer filaments. It is imperative to investigate interfacial bonding between the thermoplastic matrix and rCF in a reinforced FDM filament to understand the effect the rCF has on mechanical properties.

From the literature, studies on the effect of fiber length and fiber size in carbon fiber reinforced filament only focused on the 3D printed parts, not the filament. Consideration of the effect of these parameters on the filament, particularly rCF composite, is still insufficient. None of the studies considered other important aspects such as the surface quality and porosity of the reinforced filament. More studies are required to understand the mechanical and physical properties of thermoplastic filament under different fiber sizes and loadings. Apart from that, it is also important to investigate other physical properties of rCF-reinforced filaments, such as water absorptivity. This is to enhance the commercial viability of rCF in potential sectors, such as sport and marine applications.

This study investigates the effects of different fiber loadings (10%, 20%, and 30%) and fiber lengths (63 µm, 75 µm, and 150 µm) on the tensile properties, density, water absorptivity, and surface roughness of rCF-reinforced PLA FDM filaments.The fractured surfaces were also examined at a microscopic level using scanning electron microscopy (SEM). The findings of this study provide information to FDM users so that they are able to make informed decisions about the best strategy in using short or milled rCF for a better mechanical performance and surface quality of their products.

## 2. Materials and Methods

### 2.1. Material Spesification

**Figure 1:**
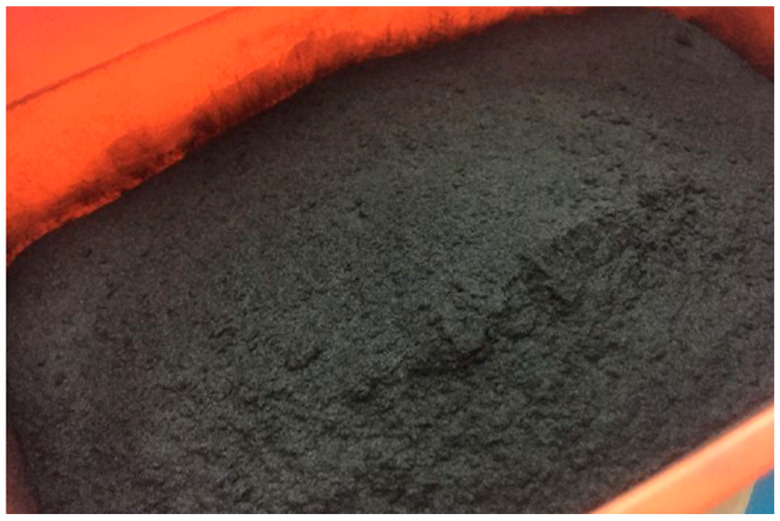
rCF recovered from a pyrolysis process.

The rCF used in this study was supplied by ELG Carbon Fibre Ltd., Coseley, United Kingdom, as shown in Figure 1. The rCF was recovered from carbon fiber composite waste via a pyrolysis process. As a post recycling step, the rCF was milled into a powder form. The PLA material was supplied by Mecha Solve Engineering (Selangor, Malaysia). The selection of this thermoplastic was based on its compatibility with the FDM process, which requires materials with a low melting point. The properties of the reinforcement fiber, as taken from the material data sheet, are listed in Table 1, alongside the PLA matrix.

### 2.2. Recycled Carbon Fiber Specification

Particle size fractions of rCF were classified using a mechanical sieving process. The sieving was carried out using a TI Motorised mechanical sieve shaker. The sieve mesh apertures were 150 µm, 75 µm, and 63 µm in diameter. Once the sieving process was completed, the quantity of each group of particles was measured using an electronic weighing balance. Material retained on the sieve was grouped into three sizes in the ranges of 63 µm, 75 µm, and more than 150 µm.

### 2.3. Fiber Length Measurement

**Figure 2:**
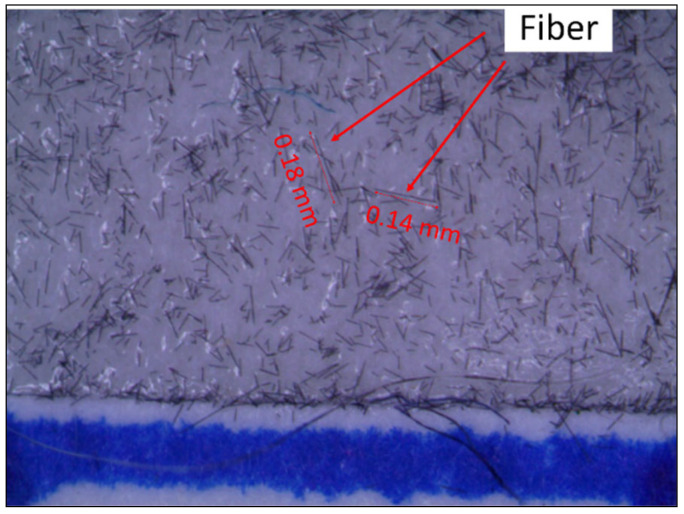
Image of rCF under an optical microscope.

Measurement of the length distribution of rCF is necessary for determining the performance of reinforcement when incorporated into a new matrix. When measuring the fiber length, double-sided sticky tapes were placed on a paper with a length scale. A batch of rCF from each group after the sieving process was randomly selected and distributed onto the sticky tapes. As displayed in Figure 2, an image of the fibers was captured using an optical microscope and transferred to a computer. The fiber length was manually measured using ImageJ software. The fiber length distribution graph was plotted for each group. Mean and standard deviation were also calculated.

### 2.4. Fabrication of Reinforced Filament

The rCF acted as a filler and was mixed homogenously with PLA pellets to produce six compositions which each weighed 200 g. The mixing was based on weight loading and thes size of the particles of rCF, as presented in Table 2. For the investigation of the weight loading effect, a constant particle-size group of 63 µm was used. For the effect of particle size, a weight loading of 10% was chosen. This choice was made based on the optimum weight loading found in previous studies by Allawi, et al. [34], Salleh, et al. [35], and Yang, et al. [18], as well as mixing efficiency during the extrusion process.

**Figure 3:**
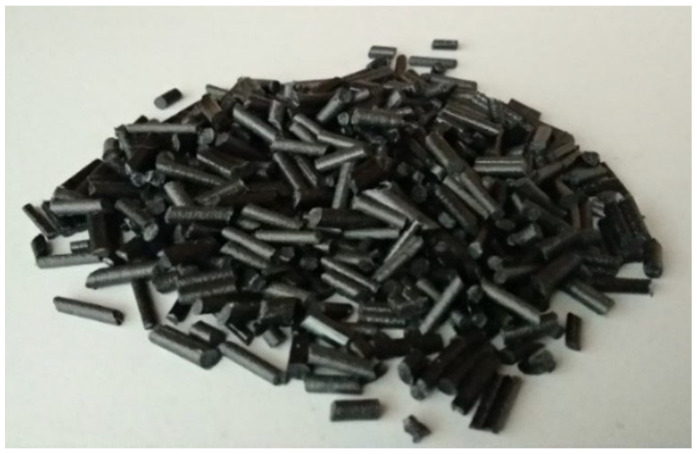
rCF/PLA pellets after the mixing and palletizing process.

The rCF and the PLA pellets were mechanically mixed using a LabTech twin screw extruder. The process began when the materials were fed into the hopper of the extruder. The speed screw was set to 160 rpm, with a feeding screw of 20 rpm at a temperature of 200 °C. After the materials were extruded, the process was continued with a palletizing process using a cutter. The rCF/PLA pellets, as shown in Figure 3, were collected in a bin.

**Figure 4:**
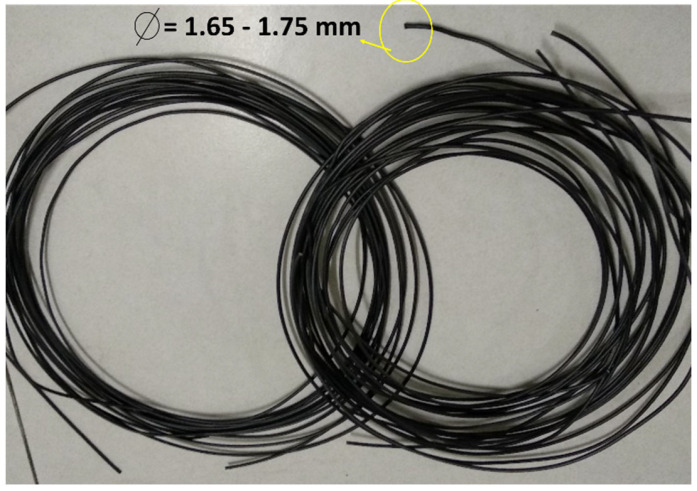
rCF/PLA filament composite.

The rCF/PLA filaments were fabricated using a Wellzoom filament extruder. The machine is a single screw extruder that converts pellets of plastic into a filament form which can be used for the FDM process. After the extruder was heated, the rCF/PLA pellets were fed into the hopper. The plastics melted and were extruded through a nozzle with a diameter of 1.75 mm. The temperature used for the rCF/PLA pellets was 200 °C. Pure PLA filament was also fabricated to act as a control product in comparison with the rCF-reinforced filaments. The resulting filaments are displayed in Figure 4.

### 2.5. Testing and Characterization of rCF Reinforced Filament

#### 2.5.1. Tensile Test

The tensile strength and elastic modulus of the filament samples were determined using a 50 kN Shimadzu Universal Testing Machine. The test used a crosshead speed of 1 mm/min. Five specimens were tested for each product, and the average value was calculated. Filament samples with a 75 mm length size were tested according to the ISO 527 standard, and the gauge length was set to 50 mm. The samples were clamped between two plates at each end. The tensile properties were calculated and recorded using Trapezium X Material Testing Software.

#### 2.5.2. Microscopy and Porosity Characterization

A TM3000 Table Top Scanning Electron Microscope was used for the scanning electron microscopy (SEM) analysis. The analysis was carried out to compare the surface conditions of rCF and vCF. For the composite filament, the characterization included fracture portions of the tensile samples. Prior to the test, all samples were mounted on aluminium stubs to undergo platinum sputtering coating to avoid surface charging. Images of the fractured samples were captured by subjecting them to a voltage of 3–5 kV and magnification up to 1000× in high vacuum mode. The fiber pull-out and porosity of the fractured sample were observed and subsequently analyzed.

#### 2.5.3. Water Absorption Test

The water absorptivity of the samples was determined based on the ASTM D570 standard. The weight of the samples before water immersion, *M_o_* was measured after they were oven-dried at 60 °C and then placed in a room at room temperature of 25 °C for 24 h. Then, the filaments were immersed in a container consisting of tap water at room temperature. Weight gain monitoring was recorded once a day for 14 days. For each type of filament, five samples were used, and an average value was calculated. Moisture content or percentage water absorptivity, Δ*W*, was determined according to Equation (1).

(1) $$\Delta W=\frac{M_{t}-M_{o}}{M_{o}}\;\times \;100$$

where *M_o_* = weight of the sample before water immersion, and *M_t_* = weight of the sample after water immersion.

#### 2.5.4. Density Test

The density of a material is defined as mass per unit volume. The test method to determine the density of the composite filament was performed according to standard ASTM D792-91. The density was calculated using the weight of the filaments measured in air and water. The density of air was assumed negligible. The density of distilled water was taken as 0.9975 g/cm^3^ at 23 °C.

#### 2.5.5. Surface Roughness Test

In determining the surface roughness of the rCF/PLA filament specimens, a Mitutoyo F-3000 surface-roughness-measuring instrument with software FORMTRACEPAK was used. The size of the contact stylus used was 0.2 mm. The sample was clamped onto the stage platform, and the stylus was located to touch the sample surface manually. The software initiated the movement of the stylus on the surface in a range of 10 mm. The arithmetic mean deviation of the surface roughness, the Ra value, was measured at three different positions in a transversal path on the sample surface. After the screen output and Ra value were displayed, the position of the same sample was changed to obtain the average value and the procedures were repeated.

## 3. Results and Discussion

### 3.1. Fiber Length Distribution

**Figure 5:**
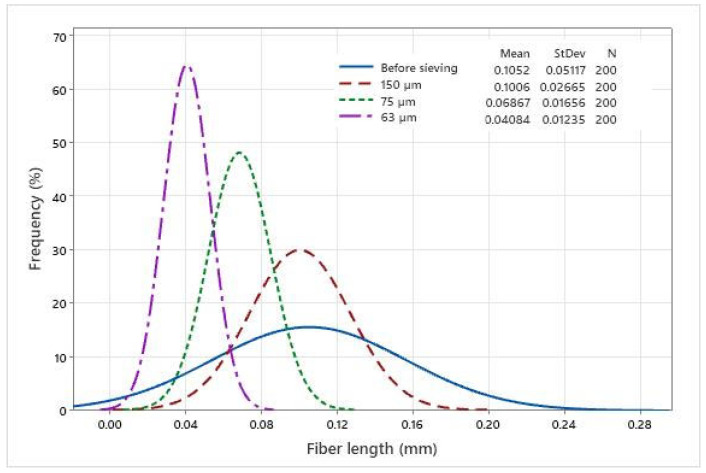
Fiber length distribution based on the size groups.

Fiber length distribution analysis was carried out to determine the efficacy of the sieving process. The distribution of different fiber sizes could affect the properties of the composite filaments. The measured rCF length distribution before and after the sieving process is plotted in Figure 5.

From Figure 5, it can be seen that the fiber length is well distributed according to the mesh sizes, compared to the fibers before sieving. It is evident from the graph that the sieving process in this study is capable of separating fibers into several size categories. A high number of fibers has a size of lower than 100 µm (0.1 mm). For the non-sieved group, the fiber is well distributed across different size groups. When analyzing the mechanical properties, fiber distribution is relevant because strength and Young’s modulus are affected by fiber size and weight.

### 3.2. Analysis of the rCF/PLA Composite Filament

#### 3.2.1. Tensile Properties

**Figure 6:**
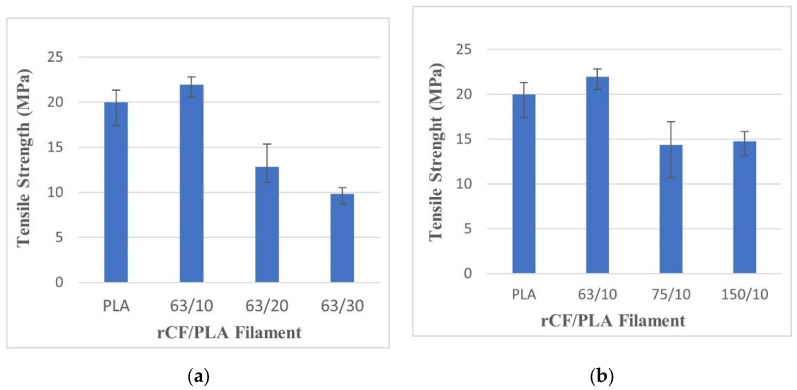
Tensile strengths of pure PLA and rCF/PLA filament based on (**a**) fiber loading and (**b**) fiber length.

**Figure 7:**
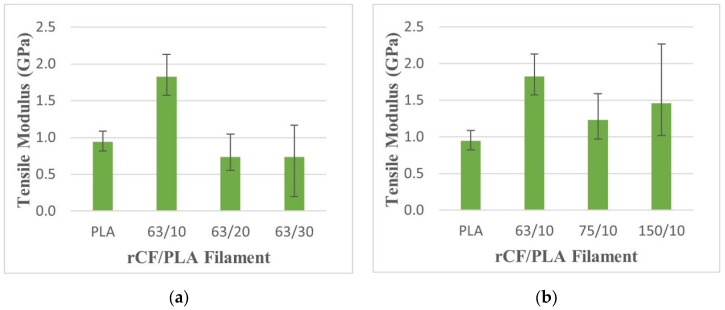
Tensile modulus of pure PLA and rCF/PLA filament based on (**a**) fiber loading and (**b**) fiber length.

**Figure 8:**
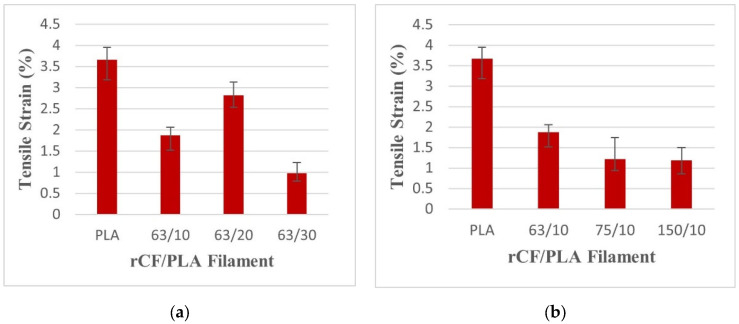
Tensile failure strains of pure PLA and rCF/PLA filament based on (**a**) fiber loading and (**b**) fiber length.

The tensile test evaluated tensile strength, tensile modulus, and elongation at failure of the rCF/PLA samples. The results are depicted in Figure 6, Figure 7 and Figure 8, respectively. The strength and ductility of the composite filament were found to be dependent on the filler weight loading. Figure 6 and Figure 7 show that the filament containing 63 µm fibers and 10 wt% loading has the highest tensile strength. However, further addition of the filler reduced the ductility of the filament. This result is in agreement with Yu, et al. [36], who reported the effect of carbon fiber concentration on sample elasticity. That study showed that the specimens with a greater carbon fiber concentration showed lower ductility.

The addition of rCF in PLA increases the individual strength of the filament composites. In Figure 6a, as the fiber loading increases above 10%, the samples exhibit a rather obvious decline in the tensile strength. This can be explained by the reduction of fiber–matrix contact, as higher fiber loading increases the fiber–fiber contact. According to the rule of a mixture of a composite, increasing fiber loading should result in the improvement of the tensile strength, if the fiber–matrix bonding interphase is pronounced.

Similar trends were reported by Ning, et al. [37], who investigated CF/ABS with different fiber contents and lengths. When exceeding a fiber weight fraction of 7.5%, the strength of the composites decreases. This is attributed to an insufficient amount of thermoplastic to bind the fiber, thus prompting poor contact between the fibers and the matrix. Further addition of the fiber weight loading will reduce the ultimate strength of the composite.

The effect of fiber length for all filaments is shown in Figure 6b. The filament containing the 63 µm fibers is relatively higher compared to the filaments reinforced with the 75 µm and 150 µm fibers. The filaments containing smaller fibers require a larger load to reach their breaking points. Good tensile strength relies more on effective stress distribution. Hence, it is expected that lower sizes of rCF lead to uniform fiber distribution within the composite product, hence better stress transfer from matrix to fiber

In Figure 7, the tensile modulus increases with the addition of 10% fiber weight loading in comparison with the control filament. Further addition of fiber weight loading leads to a poorer tensile modulus. This reduction can be linked to the brittle behaviour of the samples with higher filler loading. The effect of different fiber sizes on the tensile modulus is not significant, as shown by the overlapping error bars in Figure 7. The results agree with a finding by Aji, et al. [38], which showed that the incorporation of different lengths of natural fibers did not indicate any improvement of the modulus as result of fiber attrition.

Figure 8 exhibits the tensile strain of rCF-reinforced PLA filament composites. The tensile strain represents the elongation of the sample during the failure. From Figure 8, all filaments have a lower elongation compared to the control filament.

This result is supported by Junaedi, et al. [39] on short-carbon-fiber-reinforced polypropylene composites. That study showed that the incorporation of carbon fiber into thermoplastic improved the tensile strength and the elasticity of the composites, with the opposite reaction for the tensile ductility. This appears to have occurred due to the significant amount of fiber loading in the composite, which caused the molecular chain to be hampered. The matrix molecule chain is only achievable when there is a matrix continuity. In this study, the presence of rCF in the PLA matrix disrupted the matrix continuity, causing the premature failure of the material.

#### 3.2.2. Morphology and Porosity Analysis of the Tensile Fractured Specimen

**Figure 9:**
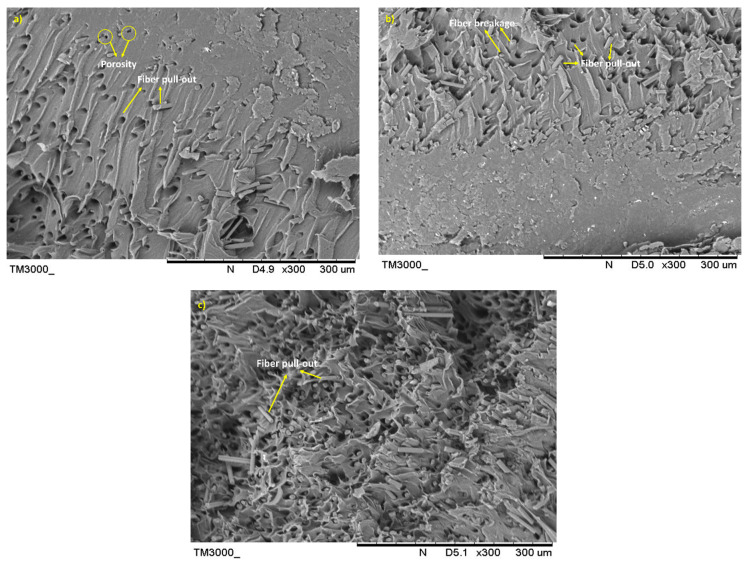
SEM images of rCF/PLA tensile fractured surface; (**a**) 63/10, (**b**) 63/20, and (**c**) 63/30 filament samples.

The SEM images of the filament composites with different fiber loading after the fracture under the tensile load are shown in Figure 9. The images show the nature of rCF and the interaction at the fracture. Closely packed interfacial bonding between the fiber and the matrix, as displayed in Figure 9a, correlated with the excellent tensile performance of the filament. The number of fiber pull-out regions is less because of strong fiber/matrix adhesion.

More fiber pull-out regions can be observed for the 20 wt% and 30 wt% samples, as indicated in Figure 9b,c, respectively. The existence of fiber pull-out is most likely due to the poor wettability and matrix dispersion of the fiber, which resulted in reduced interface bonding. At high fiber loadings, entanglement between fibers can also be the reason for a reduction in the strength of fiber holding.

**Figure 10:**
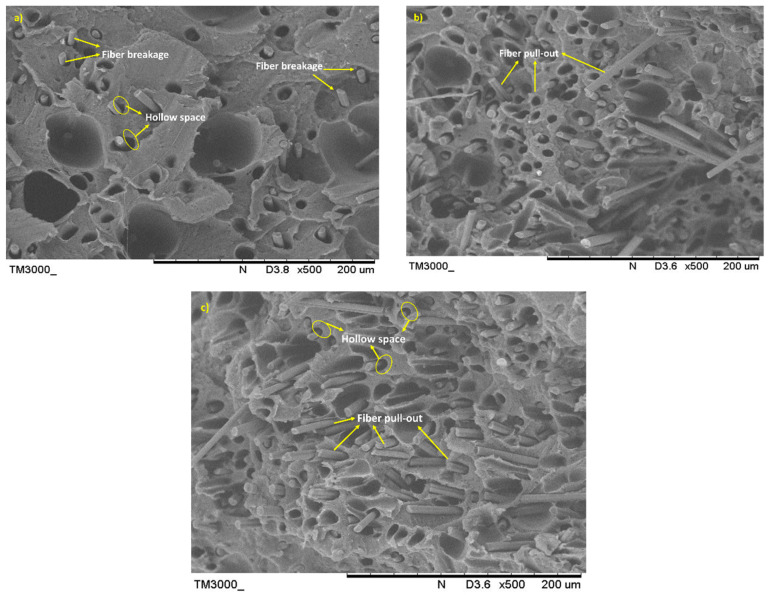
SEM images of rCF/PLA tensile fractured surface; (**a**) 63/10, (**b**) 75/10, and (**c**) 150/10 filament samples.

Figure 10 shows SEM images of the tensile fracture surface of filaments for different fiber sizes. The pull-out can be observed for the filaments with 75 µm and 150 µm fibers as shown in Figure 10b,c, respectively. The SEM micrographs in Figure 10a also reveal that the fibers are attached to the matrix, which indicates that the low viscosity PLA thermoplastic has good bonding with the fibers. Fiber breakages are noticeable in Figure 10a, explaining the strong interfacial bonding between the two constituents. For the shorter fiber, its high surface area contributes to adequate adhesion between rCF and the matrix. The pull-out regions in Figure 10b,c correlate with the reduction in the tensile strength shown in Figure 6.

The difference in the nature of the fiber and the matrix causes hollow spaces to form. The PLA material is ductile with a lower strength in nature, compared with the rCF which is more brittle. The interfacial shear stress between the rCF and the PLA matrix is usually insufficient to allow fiber breakages. The lower interfacial stress could be related to the fiber length, which, in this study, was less than the specified length required to induce a significantly greater interfacial bonding strength. Non-polar fibers are inherently incompatible with the PLA matrix during impregnation. However, the porosity has increased the permeability of the fibers, resulting in high melting-plastic uptake during the extrusion process. The internal porosity of the filaments is also indicated by the impregnated fibers, which are not uniformly dispersed in some areas.

#### 3.2.3. Water Absorptivity

**Figure 11:**
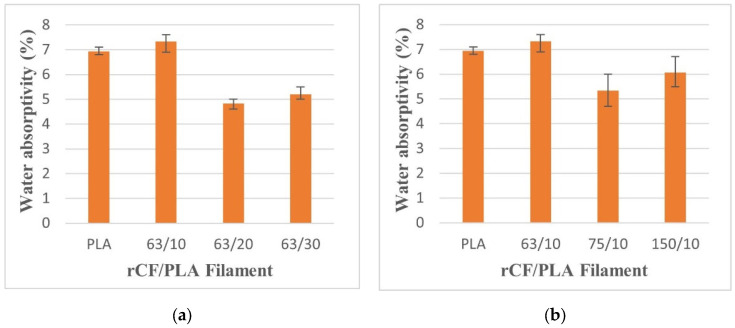
Water absorptivity of pure PLA and rCF/PLA filament based on (**a**) fiber loading and (**b**) fiber length.

Water absorptivity refers to the ability of a material to absorb moisture from its surroundings. This property is vital for a water-exposed environment or application. The water absorption level was calculated based on the weight gain relative to the dry weight of the specimens. Figure 11 presents the water absorptivity by percentage for the filament composites. The figure reveals that the filament that was 63 µm in size and had 10 wt% loading had the highest water intake, followed by the control product.

As reported by Zhai, et al. [40], carbon fiber in the composite does not absorb water when immersed in water. This is because of the non-polar nature of carbon fiber. The polar water of the molecule is unable to enter the fiber and affects its performance. The increase in the weight of composite after immersion in water is due to the hygroscopic nature of PLA, meaning that the plastics have a strong affinity to attract moisture into their internal molecular structure. When the filaments are exposed to the water, the brittle filaments will experience micro-cracking, because of the swelling of the PLA matrix. This behaviour also adds to the additional water penetration into the fiber–matrix interphase, causing stress concentration and composite failure. As more micro-cracking develops, water transfer through these cracks becomes active. Water molecules pass through the capillary fissures and continuously damage the interface, causing the fibers and the matrix to debond.

Furthermore, water absorption is also affected by the density of filaments. The higher percentage of water absorption in sample of rCF/PLA is attributed to a loosening in the packing of the filaments, which created a void in the specimens allowed more water intake. The formation of voids and spaces allows water to pass through pores in the samples. This explains that the fiber loading and the fiber length also affect the water intake of filaments when subjected to the formation of voids. From Figure 11, it can be seen that the addition of rCF can reduce the amount of water absorbed by the PLA-based products. This signifies the potential application of rCF filler in PLA matrices in a water-exposed environment.

#### 3.2.4. Density

**Figure 12:**
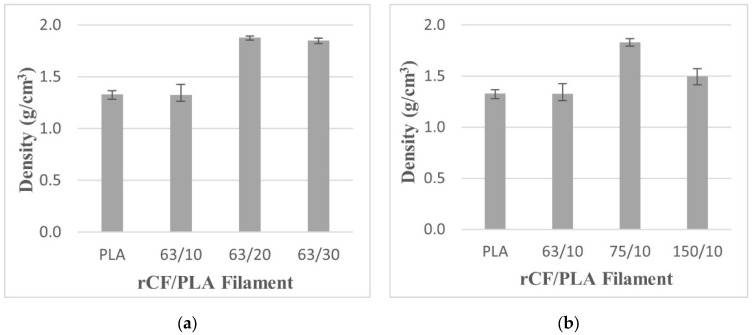
The density of pure PLA and rCF/PLA filament based on (**a**) fiber loading and (**b**) fiber length.

Figure 12 presents the density of the rCF/PLA filament composites based on fiber loading and the fiber size parameters. The filament that contained 63 µm fibers and 20 wt% loading had the highest density value at 1.87 g/cm^3^ and the lowest density was the filament that contained 63 µm fibers and 10 wt% loading at 1.30 g/cm^3^, which is almost similar to the control filament. In Figure 12b, it can be noticed that the density increases as the filler size increases from 63 µm to 75 µm, but the density reduces as the filler size further increases to 150 µm. The density also marginally reduces when fiber loading is increased from 20 wt% to 30 wt%, as shown in Figure 12a.

It can be inferred that the density value has a trade-off effect between the presence of the fibers and the voids. The lower density of filament samples is related to the presence of voids and spaces in samples. Voids are formed mainly because of air entrapment during extrusion and moisture absorbed during material storage. A low density indicates a higher void content that could cause weaker adhesion between the fiber and the matrix. This poor adhesion results in weaker interfacial strength, which reduces the strength of the filament. Comparing Figure 6 and Figure 12, the tensile strength of the filament that contained 63 µm fibers and 10 wt% loadings (63/10) is the highest despite its lower density. This can be related to a low number of pores in the filament, as shown in SEM images in Figure 9a and Figure 10a.

The increment of density for the filaments with higher loadings (20 wt% and 30 wt%) may be attributed to the addition of the fiber. However, the number of voids or porosities is also significant in those higher fiber-loading filaments, hence the deterioration of the strength of the samples. This is confirmed based on Figure 10c, which shows the presence of large amount of voids in the filament containing the 150 µm fibers. Mutual abrasion of fiber also led to fiber fracture and crack initiation due to void coalescence. Generally, the density of the filament composites is significantly influenced by both particle size and weight loading parameters.

#### 3.2.5. Surface Roughness

Apart from the mechanical properties, the surface quality of FDM-printed products is vital in achieving the specific requirements of their applications. The filament must also have a smooth surface condition, to avoid any disruption or clogged material when the filament is travelling in the machine tube and nozzle.

**Figure 13:**
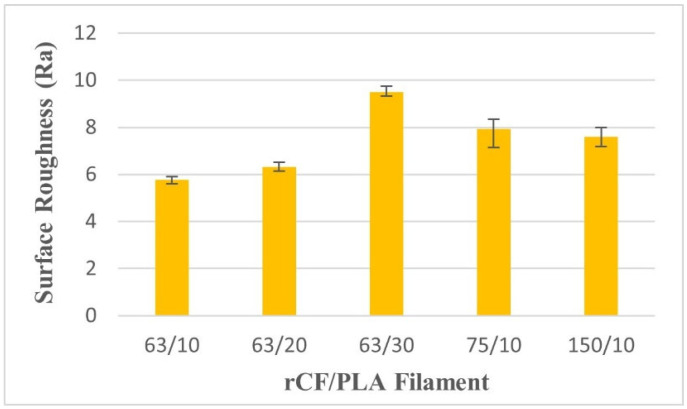
Surface roughness of rCF/PLA filaments based on fiber loading and fiber length.

In this study, surface roughness was measured using arithmetical mean roughness, Ra. The lower value of Ra indicates a smoother surface. As shown in Figure 13, the filament with 63 µm fibers and 10 wt% loadings has the lowest Ra value. The surface roughness is ascribed to the presence of rCF adhering to the surfaces. For the effect of fiber weight loading, the increase in the fiber loading of up to 30 wt% resulted in the agglomeration of excess fiber near the surface of the filament. This leads to a rougher surface. In terms of fiber size, longer fiber sizes also lead to poor surface quality as a result of the agglomeration and the possibility that the fibers did not uniformly disperse in the matrix. This finding is consistent with that of Wang, et al. [41], who reported that the surface quality of PEEK composites deteriorates as CF fiber weight volume increases from 5% to 15%. With the addition of fiber, the high viscosity of the mixture leads to a greater flow resistance, which leads to a roughened surface of the extruded filaments.

## 4. Conclusions

This study set out to analyze the effect of rCF-fiber weight loading and fiber size in the FDM filament-fabricating processes on the mechanical and physical properties of the product. This initiative is to ensure mechanical performances and quality of FDM filaments, particularly when a relatively new reinforced material can be optimized. The information can minimize challenges during the printing process, such as filament breakage and nozzle clogs. From here, properties of rCF-based filament feedstock for FDM method as well as the overall performance of the subsequent printing process can be well understood.

The results of this study indicate that the material parameters in the filament production significantly affect the properties of the composite filament. The addition of rCF with a 63 μm fiber size and up to 10 wt% into the PLA matrix increased the tensile properties of the filaments. It can be said the parameters were the best choice for excellent mechanical strength and good surface quality. The addition of more than 10 wt% rCF and the usage of fiber size greater than 63 μm caused the tensile strength and modulus to reduce. The greater rCF weight loading and size could act as a stress raiser in the filament. In terms of the water exposure condition, the presence of rCF could avoid high water intake. For a mechanical-demanding application, reinforcement with rCF is desirable in increasing the density of the product.

The findings of this study also highlight a clear trade-off between optimized properties. It is important to understand the final application of the product in order to determine which properties are important to be optimized and which can be moderated.

Overall, this study provides underpinning scientific-based research in addressing the grand challenges of reusing CF composite waste. The findings of this study contribute some knowledge about transforming waste into added-value 3D printed products with excellent mechanical performance. With the knowledge of optimized material parameters, composite users can now take a scientifically informed decision regarding the potential use of milled rCF in 3D printing processes. With a cheaper price and lower embodied energy compared to vCF, rCF could be a favourable option or substitute in the future.

## Figures and Tables

**Table 1:** Properties of rCF and PLA as provided in the manufacturer sheet.

Properties	rCF	PLA
Tensile strength (MPa)	3470	53
Elastic Modulus (GPa)	246	3.5
Tensile Strain (%)	-	6
Density (kg/m^3^)	1800	-
Carbon Content (%)	>95	-
Diameter (µm)	7	-

**Table 2:** Compositions of particle size and weight loading of rCF and PLA mixtures.

Particle Size (µm)	Weight Loading of rCF (% (g))	Weight of PLA Matrix (g)	Sample Name
63	10 (20)	180	63/10
63	20 (40)	160	63/20
63	30 (60)	140	63/30
75	10 (20)	180	75/10
150	10 (20)	180	150/10

## Data Availability

No data is available.
